# Modifying Yeast Tolerance to Inhibitory Conditions of Ethanol Production Processes

**DOI:** 10.3389/fbioe.2015.00184

**Published:** 2015-11-11

**Authors:** Luis Caspeta, Tania Castillo, Jens Nielsen

**Affiliations:** ^1^Centro de Investigación en Biotecnología, Universidad Autónoma del Estado de Morelos, Cuernavaca, Mexico; ^2^Novo Nordisk Foundation Center for Biosustainability, Chalmers University of Technology, Gothenburg, Sweden; ^3^Department of Biology and Biological Engineering, Chalmers University of Technology, Gothenburg, Sweden; ^4^Novo Nordisk Foundation Center for Biosustainability, Hørsholm, Denmark

**Keywords:** yeast, stress tolerance, cellular stress response, inhibitory environment, ethanol production process, design-based engineering, integrated -omics analysis

## Abstract

*Saccharomyces cerevisiae* strains having a broad range of substrate utilization, rapid substrate consumption, and conversion to ethanol, as well as good tolerance to inhibitory conditions are ideal for cost-competitive ethanol production from lignocellulose. A major drawback to directly design *S. cerevisiae* tolerance to inhibitory conditions of lignocellulosic ethanol production processes is the lack of knowledge about basic aspects of its cellular signaling network in response to stress. Here, we highlight the inhibitory conditions found in ethanol production processes, the targeted cellular functions, the key contributions of integrated -omics analysis to reveal cellular stress responses according to these inhibitors, and current status on design-based engineering of tolerant and efficient *S. cerevisiae* strains for ethanol production from lignocellulose.

## Introduction

Microbial fermentation of sugars from sugarcane and corn starch to ethanol is the source of around 100 billion liters of fuel ethanol annually produced in the world using *Saccharomyces cerevisiae*. There is also much interest in the use of lignocellulose as a feedstock for future production of ethanol, since this source is much more abundant and, most important, it does not compete with food for supplies (Lynd et al., 1991; Caspeta et al., 2013). However, one of the major bottlenecks for lignocellulose conversion to ethanol is that the related production processes should be economically competitive, a condition that is in detriment of yeast performance, since it must face high concentrations of toxic chemicals and harmful process conditions, for which extra operations for process conditioning to yeast tolerance are economically and energetically prohibited (Caspeta et al., 2013, 2014a). Thus, yeast cells can be exposed to inhibitory concentrations of toxic chemicals and low pH resulted from thermo-chemical pretreatment of lignocellulose. Furthermore, saccharification and fermentation of sugar polymers exposed *S. cerevisiae* to high temperatures, elevated osmolarity, and high concentrations of ethanol (Garay-Arroyo et al., 2004; Caspeta et al., 2014a). The former conditions are useful to reduce contamination and cooling efforts as well as to decrease energy utilization during downstream processing and to decrease enzyme loadings concomitant with lower production costs (Caspeta et al., 2014a).

Microorganisms capable of resisting conditions of lignocellulose ethanol production processes whereas maintaining high metabolic activity are desirable. Microbial strains with these characteristics can be isolated from natural habitats where they have been evolving these traits for a long time (Ballesteros et al., 1991; Edgardo et al., 2008; Field et al., 2015). Another option is to generate tolerant phenotypes in model organisms like *S. cerevisiae*. This requires the augmentation of limits between the relation of cellular functions and environmental fluctuations, namely to diminish the disturbing effects of inhibitory conditions on cellular functions required for growing and biofuel synthesis. Some of the targeted functions include proteome structure and stability, RNA synthesis and processing, sugar transport, membrane fluidity, and DNA processing, among others (Kültz, 2005).

Yeast and other microorganisms have gene expression and metabolic turnover programs that have been finely adjusted to improve cells fitness in the environmental fluctuations found in their natural environments (Tagkopoulos et al., 2008; Mitchell et al., 2009). Therefore, microorganisms exposed to novel environments may mount erratic non-specific responses leading them to survive or perish. Thus, one can expect that adaptation to novel environments will require the complete reprograming of cellular functions, including gene expression and metabolic turnover, which may not be attainable by multigene modification (Alper and Stephanopoulos, 2009). This is probably more evident due to the fact that stressful conditions – out of those found in the natural environments – will not be anticipated in native signaling networks. However, there is genomic plasticity that allows approaching the hypothesis that cells can acquire new functions or reconfigure macromolecular structures more suitable to new environments. The challenge then is to recognize the genomic rearrangements and the resultant levels of gene expression, according to environmental changes. In this review, we describe basic knowledge about cellular stress response (CSR) and the current strategies for improving yeast tolerance to inhibitory conditions found in lignocellulosic ethanol production processes.

## Inhibitory Conditions of Lignocellulosic Ethanol Production Process

**Figure 1:**
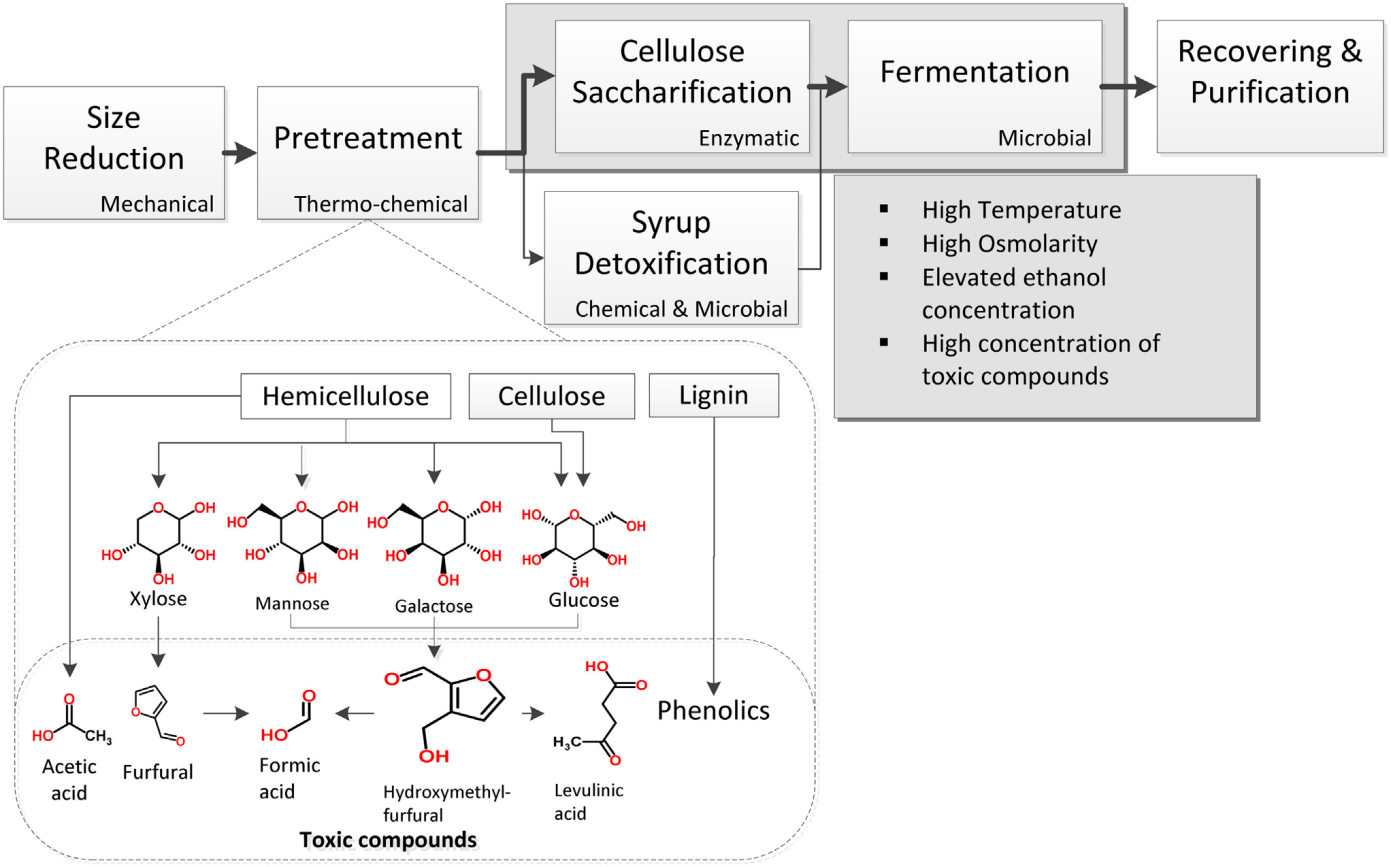
**Basic unit operations for the production of ethanol by *S. cerevisiae* using lignocellulosic biomass hydrolyzates**. Inhibitory conditions appear in pretreatment and saccharification/fermentation steps.

Lignocellulose is a tightly packed structure of the carbohydrate polymers cellulose and hemicellulose surrounded by the phenolic polymer lignin. Although several processes have been developed thus far for lignocellulose conversion to ethanol, a characteristic one includes the general steps shown in Figure 1. Once the material has been chopped in pieces, a pretreatment step mainly consisting of a thermo-chemical treatment of lignocellulose is used for its hydrolysis into fermentable sugars. These get dissolved in a syrup that can also contain acetic, formic, and levulinic acids, as well as furans and phenolic compounds released during pretreatment (Larsson et al., 1999; Palmqvist and Hahn-Hägerdal, 2000b). Since these chemicals reduce yeast growth and ethanol production (Zaldivar et al., 1999; Larsson et al., 2000; Palmqvist and Hahn-Hägerdal, 2000a), several efforts have been made to avoid their production (Palmqvist and Hahn-Hägerdal, 2000a; Caspeta et al., 2014a). Another option is to reduce their concentrations by different detoxification methods (Palmqvist and Hahn-Hägerdal, 2000a), but extra operations negatively impact energy balance and production costs (Caspeta and Nielsen, 2013).

Whatever the hydrolysis method, this must ensure syrups with high sugar concentrations. Concentrations of fermentable sugars higher than 250 g L^−1^ guarantee ethanol titers above 100 g L^−1^, required to reduce energy consumption and production costs during downstream operations (Haelssig et al., 2008). To reach these concentrations, suspensions with around 416 g of pretreated lignocellulosic biomass containing 60% of fermentable sugars – a high gravity suspension will be needed. The resulted syrup would contain high amounts of toxic chemicals as well as elevated amounts of insoluble lignin and cellulose fractions. If saccharification and fermentation of cellulose is performed simultaneously, the high gravity of cellulose/lignin suspension could impair both, enzyme activity and cell growth (Caspeta et al., 2014a). Whereas, performing saccharification and fermentation separately exposes yeast cells to toxic compounds and very high osmolarity.

Performing thermo-chemical hydrolysis at mild conditions reduces toxic compounds formation and can disrupt lignocellulose structure (Pan et al., 2006; Caspeta et al., 2014a), keeping hemicellulose and/or cellulose polymers intact for their further hydrolysis with cellulosic enzymes. Saccharification is costly and highly affected by process temperature and solid loadings (Ingesson et al., 2001; Caspeta et al., 2014a). Most of commercial enzymes have optimal temperatures higher than 45°C and the enzymes’ industry have been trying to increase it, because of operations at high temperatures are highly desirable to reduce contamination and cooling efforts. This condition, however, limits simultaneous saccharification and fermentation since most of yeast strains do not tolerate temperatures higher than 40°C.

In summary, *S. cerevisiae* can be exposed to a number of toxic compounds formed during pretreatment of biomass, e.g., low pH, unusual levels of sugar concentration and solid loadings in cellulose suspensions and hydrolyzates, lethal temperatures occurring in saccharification, and high ethanol concentrations resulting from the fermentation. All these inhibitory conditions affect cellular functions in the different forms as described below.

## Inhibitory Effects of Harmful Conditions of Lignocellulosic Ethanol Production Process

### Inhibitory Effects of Toxic Compounds

The inhibition of cellular growth and metabolism by toxic compounds formed or released during hydrolysis of lignocellulosic biomass was detailed elsewhere (Palmqvist and Hahn-Hägerdal, 2000b), and summarized in Table 1. Harmfulness of acetic, formic, and levulinic acids depends on extracellular and intracellular pH, membrane permeability, and toxicity of the anionic forms of the acids (Palmqvist and Hahn-Hägerdal, 2000b; Maris et al., 2004). Once the acid goes into yeast cell, the intracellular pH drops and excessive proton accumulation is pumped out of the cells by various mechanisms, including proton translocation with the plasma membrane H^+^-ATPase mediated by ATP hydrolysis (Holyoak et al., 1996; Maris et al., 2004). This cellular process can be very intensive in terms of ATP utilization. For example, in presence of sorbic, benzoic, and octanoic acids at pH 4.5, 5.0, and 4.0, respectively, a 10-, 4-, and 1.5-fold decrease in intracellular ATP levels can be observed due to increasing energy for maintenance of the internal pH (Viegas and Sá-Correia, 1991; Verduyn et al., 1992; Holyoak et al., 1996), with a concomitant reduction of biomass yields (Viegas and Sá-Correia, 1991; Verduyn et al., 1992). Furthermore, acetic and formic acids, in their anionic forms, are lipophobic and enter to the cell as undissociated forms, which prevail at external pH values below 4.8 (Casal et al., 1996). Inside the cell, the acid is dissociated and the intracellular pH decreases. It has been shown that intracellular concentrations higher than 120 mM of acetic acid reduce enolase and phosphoglyceromutase activities by 50% respect to non-acidic conditions (Pampulha and Loureiro-Dias, 1990). However, evidence suggests that proton exporting is the major contribution for reduced growth rate upon yeast exposition to acids.

**Table 1:** **Examples of negative effects of inhibitory conditions found in ethanol production processes on yeast performance**.

Stress	Negative effects in the yeast cells	Reference
Exposition to toxic compounds (furfural, HMF, and phenolic compounds)	Chromatin changes, DNA damage, and reduction of translation activity	Allen et al. (2010), Ask et al. (2013a)
	Enzyme inactivation	
	Reduction of the intracellular concentrations of NAD(P)H	Ask et al. (2013b)
	Negative effects on sorting and signaling functions	Keweloh et al. (1990)
	Reactive oxygen species formation	Larsson et al. (1999)
	Low biomass yields	
Exposition to organic acids	Reduction of biomass yields	Viegas and Sá-Correia (1991)
	Decrease of the intracellular ATP levels, concomitant to an increase of the maintenance energy	
	Drop of the intracellular pH	Holyoak et al. (1996)
	Reduction of enzymatic activities	Pampulha and Loureiro-Dias (1990)
Exposition to ethanol	Impairment of cellular wall permeability	Kubota et al. (2004)
	Disruption of sorting and signaling functions, with an increment of the cell size	Jones and Greenfield (1987)
	Induction of petite mutants without mitochondrial DNA (rho^0^)	Ibeas and Jimenez (1997)
	Reduction of metabolic activity	Nagodawithana and Steinkraus (1976)
	Impairment of acid resistance	Pampulha and Loureiro-Dias (1989)
Osmotic	High accumulation of glycerol	Hohmann (2002)
	Accumulation of ethanol	D’Amore et al. (1988)
	Disruption of actin cytoskeleton	Chowdhury et al. (1992)
	Disruption of MAP kinase cascade	
	Reduction of cell viability	
Physicochemical (temperature and pH)	Augmentation of detrimental effects of toxic compounds	Piper (1993), Kültz (2005)
	Modification of the protein functional structure	
	Reduction of enzymatic and metabolic activities	
	Reduction of the cell growth	

The 5-hydroxymethyl furfural (HMF) and 2-furaldehyde (furfural) are formed from thermal oxidation of hexoses and pentoses during pretreatment (Palmqvist and Hahn-Hägerdal, 2000b) – Figure 1. These compounds induce chromatin changes, DNA damage, reduced translation, and inactivation of various glycolytic enzymes (Banerjee et al., 1981; Allen et al., 2010; Ask et al., 2013a) (Table 1). Yeast can metabolize furfural and HMF to their less toxic alcohols by oxidoreductases using NAD(P)H as a cofactor, a metabolic process that occurs at high rates (Diaz De Villegas et al., 1992; Ask et al., 2013a). Their conversion increases the cellular energy for maintenance and reduces the concentration of redox cofactors (Taherzadeh et al., 1999; Sárvári Horváth et al., 2003; Ask et al., 2013a). Thus, this is associated to a reduction of glycerol production and an increase of acetate production during ethanol fermentation in the presence of furfural (Palmqvist et al., 1999a; Sárvári Horváth et al., 2003; Ask et al., 2013b). Results from exposing *S. cerevisiae* to these chemicals suggested that yeast growth is more sensitive to furfural than to HMF or high ethanol titers (Taherzadeh et al., 1999), because HMF has lower permeability and its conversion is less efficient than furfural (Larsson et al., 1999). Besides, accumulation of reactive oxygen species induced by furfural can damage *S. cerevisiae* mitochondrion and vacuole (Allen et al., 2010). Both compartments regulate redox balance of cytosol and losing their functions can result in a reduction of glucose consumption rates.

When mixtures of acetic acid and furfural are present in the fermentation, the specific growth rate decreased more than the sum of the individual effects (Palmqvist et al., 1999b), suggesting that cells expend higher amounts of energy for excreting acid anions, protons, and furfural out of the cell, as well as for reactive oxygen species formed during furfural assimilation. The growth-inhibitory effects by potential lignocellulose-derived inhibitors, including phenols [lignin, vanillin, 4-hydroxybenzaldehyde (4-HB), and syringaldehyde], furans (furfural and 5-hydroxymethyl-2-furaldehyde), and organic acids (levulinic, formic, and acetic) on the growth and ethanol production were investigated. From these, phenols and furans exhibited potent inhibitory effects at a concentration of 1 g L^−1^, while organic acids had insignificant impacts at concentrations of up to 2 g L^−1^.

Phenolic compounds released from the hydrolysis of lignin are poorly soluble in aqueous solutions and they can be incorporated into cellular membranes where their partition is higher (Heipieper et al., 1994). Here, phenolic compounds mainly interfere with proteins function and trigger changes in the protein to lipid ratio (Keweloh et al., 1990). Hence, these compounds affect cellular functions like sorting and signaling, as well as cause membrane swelling. Among the 13 tested phenolic compounds, the 4-hydroxy-3-methoxycinnamaldehyde is the most toxic (Adeboye et al., 2014). This, vanillin and catechol are major constituents of syrups from pretreated lignocellulose (Ando, 1966; Palmqvist and Hahn-Hägerdal, 2000b). It is also abundant in hydrolyzates of hardwood, which is toxic at concentration of 1 g L^−1^, reducing 30% of ethanol yield (Ando et al., 1986). The toxicity of phenolics is very variable as it depends on the functional groups (Ando et al., 1986; Jonsson et al., 2013; Adeboye et al., 2014); more methoxy groups are related to high hydrophobicity and toxicity (Klinke et al., 2004). *S. cerevisiae* can assimilate many of phenolics which can be part of the detoxification process occurring during fermentation (Mills et al., 1971; Delgenes et al., 1996; Larsson et al., 2000).

### Inhibitory Effects of High Ethanol Concentrations

One of the main advantages of *S. cerevisiae* for ethanol production is the high tolerance that this yeast shows respect to other microorganisms. For example, whereas *Escherichia coli* and *Zymomonas mobilis* have maximum tolerances around 60–127 g L^−1^ (Lee et al., 1980; Yomano et al., 1998), *S. cerevisiae* can tolerate ethanol concentrations up to between 115 and 200 g L^−1^ (Luong, 1985). However, ethanol concentrations higher than 150 g L^−1^ can be required to reduce costs in downstream operations. High concentrations of alcohols like ethanol and butanol impaired cellular wall permeability disrupting sorting and signaling functions, as well as provoked an increase in cell size which caused a cell cycle delay (Jones and Greenfield, 1987; Kubota et al., 2004) (Table 1). This correlates with a dispersion of the F-actin cytoskeleton, which is probably regulated by the protein kinase SWE1, which regulates the G2/M transition, since its mutations abolish this phenotype (Kubota et al., 2004). Ethanol also induces petite mutants without mitochondrial DNA (the rho^0^ mutants) and changes in mitochondrial genome (Ibeas and Jimenez, 1997; Chi and Arneborg, 1999). In combination with high temperature, ethanol exacerbates inactivation of some enzymes, for example, the alcohol dehydrogenase (ADH) and the hexokinase (Augustin et al., 1965; Nagodawithana and Steinkraus, 1976; Chen and Jin, 2006). The uptake of alanine, proton efflux, and fermentation rates can decrease when cells are exposed to 2M of ethanol (Mishra and Prasad, 1989). Disruption of proton efflux also impairs acid resistance (Brown and Oliver, 1982; Sá-Correia and Van Uden, 1983; Gao and Fleet, 1988; Pampulha and Loureiro-Dias, 1989; Aguilera et al., 2006), since this affects proton outtake for regulation of internal pH. Interestingly, the activity of β-glucosidase, a cellulosic enzyme used in saccharification, increased with increasing ethanol concentrations from 1 to 9% (v/v) (Chen and Jin, 2006). Since cellular wall is the key ethanol target, yeast changes lipid composition, incrementing the proportion of polyunsaturated fatty acids (FAs), ergosterol, and phosphatidylcholine (Mishra and Prasad, 1989; Kajiwara et al., 1996; Chi and Arneborg, 1999). This response is also observed in thermal stress. Eventually, moderate ethanol concentrations also reduce water activity with consequences in metabolic activity (Hallsworth, 1998).

### Inhibitory Effects of High Osmolarity

High gravity fermentations are required for economic considerations. Glucose concentrations superior to 300 g L^−1^ are needed to reach ethanol titers higher than 150 g L^−1^. Thus, the osmolarity of a hydrolyzate can be of 20–200 g L^−1^ of salt (0.6–8.6 Osm) (Olsson and Hahn-Hägerdal, 1993). *S. cerevisiae* can resist 4 Osm, which is much higher compared with *Z. mobilis*, which resists until 1.2 Osm. After being exposed to high osmolarity, yeast cells accumulated high amounts of glycerol which serves as an osmolyte (Hohmann, 2002). Osmotic shock disrupts actin cytoskeleton and invaginations appear affecting the conformation of actin bundles that disturbs MAP kinase cascade, which regulates cell cycle (Chowdhury et al., 1992). This also causes water to flow out of the cell, increasing the concentration of cellular components, including ion concentrations that can serve as a sensor for cellular signaling pathways (Hohmann, 2002). Under osmotic pressure, the excretion of ethanol and glycerol is impaired, leading the accumulation of intracellular ethanol and a decrease in cell viability (Panchal and Stewart, 1980; D’Amore et al., 1988). It seems that membrane fluidity is less prone to be affected by high osmolarity since medium pH does not have a significant effect on yeast growth at high glucose concentration, but only on ethanol accumulation (Narendranath and Power, 2005).

### Inhibitory Effects of High Temperature

Temperature pervasively practically affects all cellular macromolecules and metabolic functions (Table 1). Increasing temperature from 25–28°C to 40°C caused a substantial reduction of protein synthesis (Lindquist, 1981; Hottiger et al., 1987), which is accompanied by increasing trehalose accumulation (Hottiger et al., 1987; Neves and Francois, 1992). Both responses are essential to acquire thermotolerance (De Virgilio et al., 1994; Singer and Lindquist, 1998), since null mutants in the trehalose synthase (*TSL1*) are more sensible to thermal stress (De Virgilio et al., 1994) and significantly decrease heat-shock genes transcription (Hazell et al., 1995), while cells carrying *CYR1-2* mutation produce trehalose constitutively, and are significantly more tolerant than the wild type (Hottiger et al., 1989). There is also evidence that trehalose catabolism is needed to acquire thermotolerance and recovering of cellular homeostasis from thermal shock upon temperature upshift from 30 to 40°C (Nwaka et al., 1994, 1995). This is evidenced by the recovery of protein production and bud formation after starting trehalose degradation (Hottiger et al., 1987). Trehalose accumulates simultaneously with a reduction of glycolytic rates, albeit intracellular glucose concentrations remain constant (Neves and Francois, 1992). Decreasing of glycolytic rates corresponded to lower activity of the Ras/cAMP pathway upon thermal shock, which favored trehalose synthesis in detriment of glucose catabolism and cells growth (Shin et al., 1987; Neves and Francois, 1992; Piper, 1993; Tokiwa et al., 1994). After recovering homeostasis, cells increase Ras/cAMP pathway activity and glycolytic fluxes (Piper, 1993). Both circumstances seem to regulate cyclins activity (CLN1, CLN2, and CLN3) and transcription of *CLN3*, which are required for cell cycle progression at the START point in G1 phase (Tokiwa et al., 1994; Shi and Tu, 2013), following bud formation. It was recently shown that accumulation of acetyl-CoA, a central metabolite from glucose catabolism, triggers histone acetylation and transcription of *CLN3* (Shi and Tu, 2013).

During the acquisition of thermotolerance, yeast cells also change the lipid composition of cellular membrane. Temperature increment caused the increase of saturations and length of FAs as well as a reduction of FA composition in membranes (Suutari et al., 1990, 1997). The synthesis of long-chain bases (LCBs), which are important for membrane fluidity and dynamics, and with possible role in the regulation of signal transduction pathways, also increased (Dickson et al., 1997; Jenkins et al., 1997). Changes in the synthesis of these lipids and some sterols upon temperature increase suggest that pathways supporting signaling networks of the cell wall integrity are involved in heat-shock response (Kamada et al., 1995; Verna et al., 1997; Imazu and Sakurai, 2005). Overexpression of genes coding for antioxidants and enzymes involved in carbon metabolism mediated by the stress-responsive transcription factors (TFs) MSN2 and MSN4, but the Ras/cAMP/PKA signaling pathway cAMP had a negative effect on the induction of the MSN2/MSN4 regulon (Boy-Marcotte et al., 1999; Imazu and Sakurai, 2005). Hence, the former could mainly occur in the precondition effect of trehalose accumulation.

Collectively, these results suggest a toxicity model in which inhibitory conditions associated with ethanol production processes mainly affect cellular membrane concomitant with exchange reactions between the intracellular and extracellular environment, e.g., protons/ions exchange. Accumulation of toxic chemicals through the pretreatment and fermentation operations eventually exacerbates energy requirements and the cell’s effort to maintain gradients and to continue the excretion of toxic chemicals. Although mild pretreatment operations or incorporation of detoxification processes reduce the concentration of toxic compounds, such options have to be carefully considered as it may increase the costs and energy consumption. Cellular gradients can also be maintained by increasing medium pH or supplementing with specific salts. Adaptation of the yeast to process conditions through heritable modifications is, however, the ideal solution, and for this, it is necessary to understand the basic molecular mechanisms underlying the stress response in yeast.

## The Cellular Stress Response in Yeast

Organisms have developed strategies to mount stress responses to recover the constancy of internal state (homeostasis) upon being exposed to environmental changes (Tagkopoulos et al., 2008; Mitchell et al., 2009). These responses are associated to damage in cellular macromolecules and/or redox potentials which disrupt cellular functions (Kültz, 2005; Gibney et al., 2013). Thus, the CSR is universal and have a define set of targeted cellular functions including cell cycle control, protein chaperoning and repair, DNA and chromatin stabilization and repair, cellular membrane stabilization and repair, removal of damaged proteins, and some aspects of metabolism (Kültz, 2005). This assumption raised from the analysis of around 300 highly conserved proteins among different organisms including human, yeast, eubacteria, and archaea, from which more than 44 proteins change their abundance upon stress exposition (Kültz, 2003). Here, it has been pointed out, based on recent results, that most of the inhibitory mechanisms target cellular membrane, redox potentials, and exchange reactions functions.

**Figure 2:**
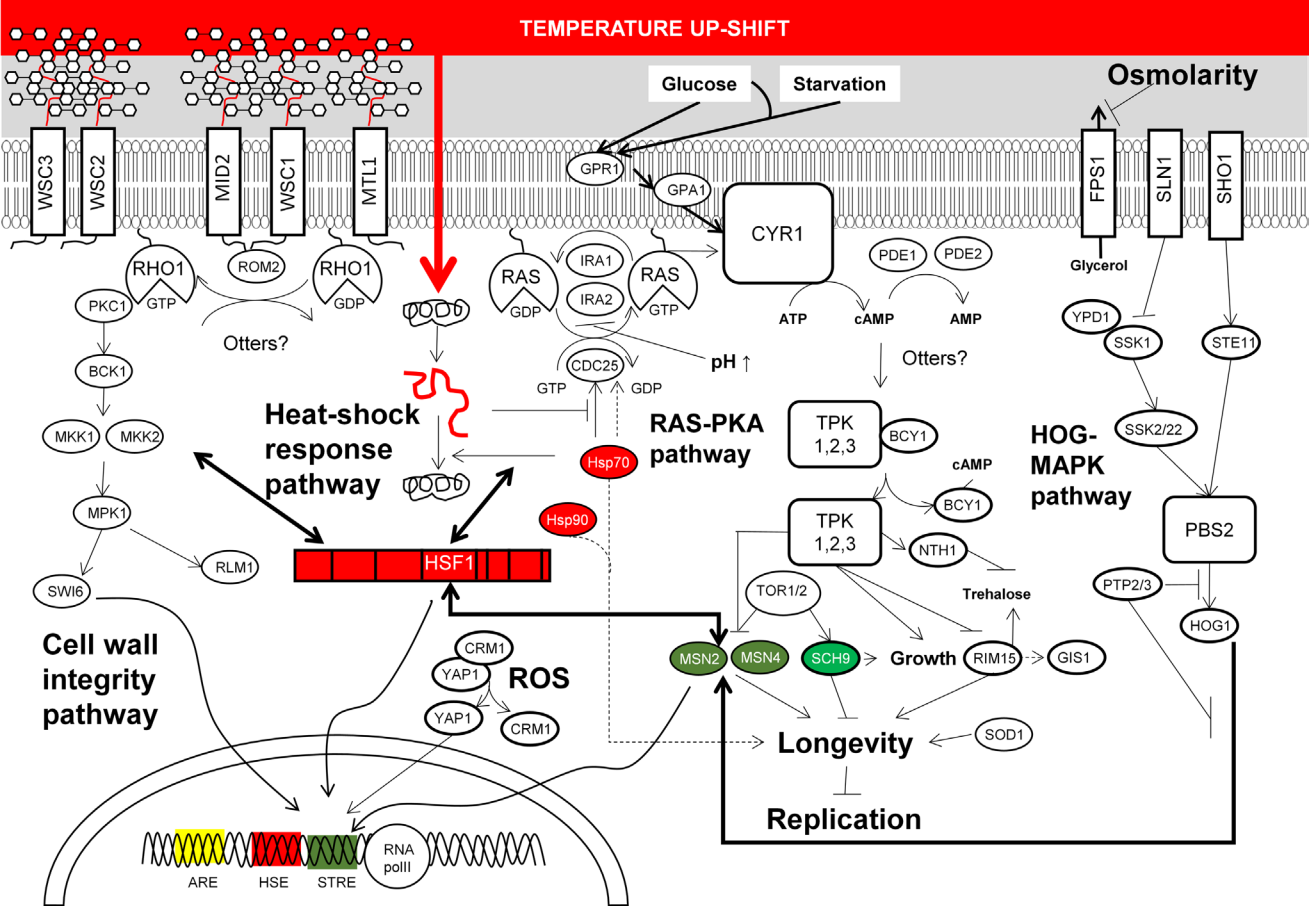
**Summarized molecular responses of *S. cerevisiae* upon exposition to chemical and physical stresses discussed in the main text**. This figure condenses the complexity of yeast stress responses upon exposition to high temperature, elevated osmolarity, and low pH. Most of these responses are also triggered upon exposition to toxic chemicals referred to in the main text. The signaling networks from membrane sensors to transcription factors which end with the reconfiguration of transcriptional programs according to stress, and the cross-talk between cellular stress responses are also depicted.

The results from transcriptomic and proteomic analyses altogether suggest that CSRs in *S. cerevisiae* overlap at specific stress conditions, whereas some responses are stress specific (Figure 2). Remarkably, this flexibility allows the coordination of stress responses according to a serial of ordered events, which are naturally organized according to its habitat. For example, yeast mounts a stress response to heat, which also serves to tolerate the stress imposed by ethanol and an oxidative environment. This could be a consequence of the domestication of yeast as these stresses appear in this order during the wine production processes (Mitchell et al., 2009). Yeast also triggers a general stress response to survive exposure to several different types of stress. Thus, when *S. cerevisiae* is exposed to environmental perturbations including temperature increase, nutrient depletion, addition of oxygen peroxide, starvation and stationary phase, DNA damaging agents, and hyperosmotic stress among others, a set of around 900 genes showed similar changes in their expression (Gasch et al., 2000). Functional analysis of these genes showed similar targeted functions than those found in proteome analysis (Kültz, 2003, 2005). Differentially expressed genes include those encoding for proteins involved in the RAS-cAMP signaling pathway, which regulate cell metabolism and cell cycle progression in response to nutrient availability (Broach, 1991; Gasch and Werner-Washburne, 2002). Simultaneously, transcription of genes involved in the CSR is also modified by the action of a set of TFs including MSN2/4, YAP1, HSF1, RLM1, and SWI6. Activation of these TFs occurs through phosphorylation cascades triggered by structural changes in proteins located in the cellular membrane; this seems to be the major target for stress agents and the origin of signaling pathways for response to stress (Figure 2).

Reprograming of gene expression in yeast is mainly governed by the general stress response TFs MSN2 and/or MSN4 targeting around 180 genes in response to thermal stress and oxygen peroxide (Gasch et al., 2000). MSN2/MNS4 also induces similar stress responses when yeast cells are exposed to other stresses, suggesting that these TFs induce a general response to various environmental changes (Causton et al., 2001). With regard to the cellular response to oxidative stress, the yeast basic leucine zipper (bZIP) TF YAP1 is mainly in charge of regulating the expression of genes associated to this stress (Temple et al., 2005), as well as in response to xenobiotic insults, including drugs and heavy metals (Lushchak, 2011). Remarkably, transcription of some genes targeted by oxidative stress is also activated by TFs MSN2/MSN4 in response to heat-shock (Gasch et al., 2000). Nonetheless, this response is mainly controlled by the heat-shock TF HSF1, which directs the expression of around 150 genes (Hahn et al., 2004). This TF also triggers gene expression changes upon starvation (Hahn and Thiele, 2004), as well as controls the expression of genes associated to life span extension (Shama et al., 1998). Furthermore, it regulates cellular wall remodeling in response to thermal and oxidative stresses (Imazu and Sakurai, 2005; Yamamoto et al., 2007). Some other relevant aspects of particular regulation of CSRs to stressors found in ethanol production processes are given below.

### Yeast Responses to Inhibitory Compounds

Metabolic and molecular responses of yeast exposition to inhibitory compounds, such as furfural and HMF, caused changes in expression of around 886 genes (Ask et al., 2013a). Functional examination of proteomic and transcriptomic analyses showed that genes involved in redox balance, oxidative and salt stress as well as the TFs, MSN2/MSN4, YAP1, and HSF1 were mostly involved in stress responses and specifically, overexpression of *YAP1* and *MSN2* were related to the increase of yeast tolerance to furfural and HMF (Lin et al., 2009; Sasano et al., 2012). Activity of the MAPK signaling pathway of the yeast response to cell wall integrity was also found to increase yeast tolerance to HMF (Larsson et al., 1999). In agreement with the transcriptional analysis in reference (Dickson et al., 1997), proteomic analysis also showed that redox and energy metabolism are significantly targeted by the stress response in yeast exposed to hydrolyzates containing furans, acids, and/or phenolics (Lin et al., 2009; Lv et al., 2014). In the last study, the authors observed differential expression of around 200 genes, a number similar to the 103 and 227 differentially expressed genes observed from yeast exposition to furfural and acetate, respectively (Li and Yuan, 2010). In this study, it was found that tolerance to furfural also required the overexpression of genes involved in the oxidative stress response, such as *SRX1*, *CTA1*, and *GRX5* as well as the *HSP78*, which encodes a mitochondrial chaperone needed for the thermotolerance of this organelle (Heer and Sauer, 2008). In addition, overexpression of some genes related to the lipid and carbohydrate metabolism have been observed within these genes. Interestingly, proteins involved in the TCA cycle were upregulated whereas enzymes of glycerol synthesis were downregulated (Lin et al., 2009). The later results strongly suggest an increment of NADH demand for furans conversion to alcohols and that this reducing power is generated in TCA cycle.

### Yeast Responses to Thermal, Ethanol, and Osmotic Stresses

In response to heat, *S. cerevisiae* typically shows transcriptional changes in genes encoding metabolic enzymes (e.g., hexokinase, glyceraldehyde-3-phosphate dehydrogenase, glucose-6-phosphate dehydrogenase, isocitrate dehydrogenase, and ADH), antioxidant enzymes (e.g., thioredoxin 3, thioredoxin reductase, and porin), molecular chaperones and their cofactors (e.g., *HSP104*, *HSP82*, *HSP60*, *HSP42*, *HSP30*, *HSP26*, *CPR1*, *STI1*, and *ZPR1*), and the TFs (e.g., *HSF1*, *MSN2/4*, and *YAP1*), among others (Lindquist, 1986; Piper, 1993; Kim et al., 2013). Most of these genes also change their expression in response to ethanol and high osmolarity (Gasch et al., 2000; Gasch and Werner-Washburne, 2002). However, stress-specific changes in gene expression also responded solely to either YAP1 or MSN2/4 (Gasch et al., 2000). In addition to the implication of TFs in the regulation of gene transcription upon exposure to different types of stress, another intriguing fact is that the dissagregase protein HSP104 and the negative regulator of the H(+)-ATPase, the HSP30 are overexpressed upon exposition to ethanol, heat, and high osmolarity (Sanchez et al., 1992; Piper et al., 1997; Kültz, 2005). These proteins are implicated in the recovery of aggregated proteins and prevent the cells from excessive energy consumption.

Cellular signaling networks of growth and stress response are antagonist. The RAS-PKA pathway, which regulates yeast proliferation in response to nutritional sensing, negatively regulates the activity of the stress-responsive elements (STRE) and the heat-shock elements (HSE) targeted by both RIM15 and MSN2/4, and HSF1 and MSN2/4, respectively (Roosen et al., 2005). Thus, high activity of the RAS-PKA pathway caused by deletion of the *BCY1* is in detriment of stress responses, whereas deletion of *RAS2* increased yeast resistance to various stresses except high temperature and osmolarity (Ruis and Schüller, 1995) – this is due to the fact that trehalose metabolism is regulated by NTH1, which is probably activated by the RAS-PKA pathway. High activity of this pathway reduces RIM15 activity, which controls the entry into the G0 phase of cell cycle in response to glucose limitation at the diauxic shift. Its regulon includes gene clusters implicated in the adaptation to respiratory growth, including oxidative stress genes (Cameroni et al., 2004). TFs RIM15, GIS1, and MSN2/4 exerts control on genes required for adaptation to oxidative and thermal stress (Cameroni et al., 2004). High RAS-PKA activity favors the activity of SCH9 kinase, which regulates ribosome biogenesis and translation initiation. This is a major target of TORC1 phosphorylation cascade, transiently reduced upon application of osmotic, oxidative, or thermal stress (Urban et al., 2007). Under favorable conditions, TORC1 promotes growth and antagonizes stress response programs (De Virgilio et al., 1994; Jacinto and Hall, 2003). Thus, TORC1 activity is reduced upon stress apparently by its sequestration in granules (Takahara and Maeda, 2012).

The RAS–PKA pathway also connects with the cell wall damage response. PCK1 and the upstream protein elements ROM2 and MTL1 of the PKC1–MAPK cell integrity pathway are needed for actin organization, and required for cellular responses to oxidative, osmotic, and heat stresses (Kamada et al., 1995; Vilella et al., 2005). More evidences on this fact were provided by two different research groups which discovered that the sensitivity to high osmolarity in the HOG-MAPK pathway mutants was reduced at elevated temperature, suggesting that the activation of the cell wall integrity pathway is mainly due to increased temperatures (Alonso-Monge et al., 2001; Wojda et al., 2003). These two pathways and the SVG pathway ensure a proper response of cell wall integrity. The latter is activated by the SHO1 sensor, which also regulates HOG signaling (Figure 2). Furthermore, it was found that membrane sensors WSC1, WSC2, and WSC3 restored the thermo-sensible phenotype of *RAS1* mutants – WSC triple mutants did not growth at 37°C (Verna et al., 1997), which is another evidence for the connection between RAS-PKA signaling cascade and the cell integrity pathway. Signal transduction of this pathway begins with the cellular membrane proteins WCS1-3, MID2, and MTL1, among others (Rodicio and Heinisch, 2010). Finalizing with the phosphorylation of the TF SWI6 leading its localization into the nucleus required for the unfolded protein response (Scrimale et al., 2009).

Thermal stress also has an important effect on the metabolic responses – e.g., glucose and oxygen consumption rates and biomass yields. In nitrogen-limited chemostats, glucose consumption rate increased up to 1.8 times at 38°C compared to 30°C (Postmus et al., 2008). Besides ethanol production rate increased 1.7 times, its yield decreased 0.6 times. Furthermore, in the cultivations at 38°C, glycerol was accumulated at 1.3 mmol g_DCW_^−1^ h^−1^ but no accumulation was observed at 30°C (Postmus et al., 2012). Despite oxygen uptake rate increase 1.1 times at high temperature, respiratory quotients (RQs) of 2.6 and 3.8 were calculated for the fermentations at low and high temperatures, respectively (Postmus et al., 2012). In the same work, a drastic drop of biomass yield was observed in cultivations grown at high temperature (38°C) as compared to the cultures developed at low temperature (Postmus et al., 2008). This behavior correlated with an increased flux of glycerol and ethanol at 38°C – these were not observed at 30°C. In both cases, oxygen consumption rate slightly increased suggesting that reducing power produced in glycolysis is balanced by glycerol production and interrupted electron transport chain.

These results altogether show the complexity of cell stress responses and the difficulties for generating complex thermotolerant phenotypes. Therefore, selection of thermotolerant microorganisms from harsh environments similar to those found in ethanol production process is still a recurrent option. However, these microorganisms will eventually be useless when process conditions change – this will be especially true in the foundation of lignocellulosic biorefineries. Thus, a rational cell design based on knowledge of cell responses will enable the design of generic cell factories that can be used in several different processes. One must be aware that there would be physical components that limit biological augmentation. In this case, synthetic biology approaches (Alper and Stephanopoulos, 2009) and utilization of additional operations to remove toxic molecules can be of interest. In the last section, we review the current strategies for rational improving of yeast tolerance to ethanol production processes.

## Improving Yeast Tolerance to Inhibitory Conditions Found in Ethanol Production Process

Some of the methods for increasing yeast tolerance to harmful conditions found in ethanol production processes are summarized in Table 2. These methods include the adaptive laboratory evolution (ALE), which is performed by serial dilution of microbial population in fresh media, maintaining or increasing the intensity of the stress (Elena and Lenski, 2003). The main advantage of this method is that increased fitness can be followed during the evolution, and populations can be screened for a strain with a useful phenotype – e.g., improved growth or increased glucose consumption. When combined with partial or complete genome sequence of isolated strains/populations, as well as genome level analysis of gene expression and metabolic fluxes, this procedure is very powerful to get basic knowledge about cell strategies that arise with the better performance (Hong et al., 2011; Caspeta et al., 2014b).

**Table 2:** **Some examples of the strategies to improve yeast stress tolerance to inhibitory conditions during the conversion of lignocellulosic biomass to ethanol**.

Molecular strategies	Specific cases	Reference
Adaptive evolution	The industrial *S. cerevisiae* ethanol red was subjected to a long-term adaptive laboratory evolution. The resultant strain was able to grow and produce ethanol using non-detoxified spruce hydrolyzates	Wallace-Salinas and Gorwa-Grauslund (2013)
	Using adaptive laboratory evolution/visualizing evolution at real time, seven strains were isolated due to their improved tolerance to lignocellulosic biomass hydrolyzates	Almario et al. (2013)
	*S. cerevisiae* strain CENPK113-7D was evolved in laboratory and seven thermotolerant strains were isolated. These strains were able to grow at 40°C under fully aerobic conditions with improved kinetic parameters as compared to the parental strain	Caspeta et al. (2014b)
Reprograming gene expression	Mutagenesis of the transcription factor SPT15 allows to increase osmotic and ethanol tolerance, improving ethanol production	Alper et al. (2006)
Direct evolution – DNA shuffling technology	The strain *S. cerevisiae* R57 was isolated after five rounds of DNA evolution by DNA shuffling. It was able to survive, grow, and produce ethanol using a substrate, hardwood spent sulfite liquor. This strain also increased its viability in presence of salt, peroxide, acetic acid, and sorbitol	Pinel et al. (2011, 2015)
Random mutations (by chemical agents or UV)	Three S*. cerevisiae* strains with improved tolerance to vanillin, furfural, and acetic acid were generated by random mutagenesis with ethyl methane sulfonate coupled to an adaptive laboratory evolution strategy	Shen et al. (2014)
Heterologous expression	The laccase I from *Trametes sp*. was cloned in yeast. The laccase-1 displaying yeast has oxidation activity and was effective during pretreatment for ethanol fermentation	Nakanishi et al. (2012)

Evolution of linear DNA fragments upon recombination of blocks of sequences rather than point mutagenesis alone has shown to be more important during evolution. The DNA shuffling technology is a procedure for rapid propagation of beneficial mutations in a direct evolution experiment (Stemmer, 1994). This is based on repeated cycles of point mutagenesis, recombination, and selection allowing molecular evolution of complex sequences, through increasing the size of DNA library (Zhang et al., 2002). In combination with cellular mating, this technology has led the generation of cells resistance to ethanol production processes (Pinel et al., 2011).

Despite changes on expression of a single gene have given good results in generating the tolerant phenotype (Caspeta et al., 2014b; Lam et al., 2014), this does not typically occurs since the tolerance to stressors requires changes of expression for thousand genes (see The Cellular Stress Response in Yeast). Therefore, the engineering of global transcription machinery has been developed to generate TFs that may lead with a proper reprograming of gene transcription network, which arises with the desired tolerant phenotype (Alper and Stephanopoulos, 2009). This method consists on the mutagenesis of TFs acting with a desired promotor sequences – e.g., the TATA-binding, and the selection of dominant mutations conferring the desired tolerant phenotype.

Random mutagenesis with chemical or physical agents, for example, the dimethyl sulfate (DMS) and UV radiation, has been used for long time to generate populations with a set of mutations from which the useful ones are selected from experiments with the desired environmental pressure. This method has been used in combination with cellular shorting procedures to analyze thousands of phenotypes and came up with the most desired one (Huang et al., 2015). Despite this method being useful for generating tolerant phenotypes, it rarely permits the analysis of mutations that arise with the desired phenotype.

### Increasing Ethanol Tolerance

Despite *S. cerevisiae* showing high ethanol tolerance, there have been many efforts to enhance this trait and generate strains tolerant to higher concentrations; here are some of the recent advances. Comparison of gene expression among tolerant and non-tolerant strains has served to recognize target genes involved in ethanol tolerance. Some genes involved in this feature are the global TF MSN2, some genes of the cAMP-PKA signaling pathway, genes related to the cellular wall integrity, and some genes encoding enzymes of lipids and carbohydrates metabolism (Lewis et al., 2010). It was shown recently that the manipulation of ions transport systems can also improve ethanol tolerance. For instance, changing potassium ion and proton electrochemical forces can improve yeast tolerance to ethanol (Lam et al., 2014). Overexpression of the *TRK1* gene, a member of the potassium transport system, and the H(+)-ATPase gene, *PMA1*, in laboratory strains increased ethanol production by around 30% respect to the laboratory strain S288C and by 10% compared to industrial strains (Lam et al., 2014). In contrast to those findings, thermally evolved *S. cerevisiae* strains, which showed slight increase of ethanol tolerance, did not overexpress *PMA1* (Caspeta et al., 2014b). The negative regulator of the H(+)-ATPase pump, the gene *HSP30*, however, increased upon thermal stress (Piper et al., 1997; Meena et al., 2011), suggesting that thermal adaptation may optimize ATP usage for proton excretion, thus decreasing energy for maintenance. Thereafter, electrical potential and proton fluxes can decrease free energy of ATP hydrolysis for proton export (Maris et al., 2004), enhancing the resistance to alcohols (Lam et al., 2014).

Transcription reprograming of yeast gene expression using the global transcription machinery engineering approach leaded with higher ethanol resistance. The mutagenesis of the TF SPT15 allowed the selection of the SPT15-300 TF with a mutation in the phenylalanine (Phe^177^ Ser) as the dominant mutation which provided increased tolerance to elevated concentrations of glucose and ethanol, as well as improved ethanol production (Alper et al., 2006).

### Increasing Tolerance to Toxic Compounds

Adaptive laboratory evolution has been successfully used for selection of yeast strains tolerant to lignocellulose hydrolyzates containing furfural, HMF, and acetate (Liu et al., 2005; Keating et al., 2006; Heer and Sauer, 2008). Evolution of yeast populations in synthetic medium containing 3 mM furfural resulted in the selection of tolerant strains after 300 generations (Heer and Sauer, 2008). These strains reduced the lag-phase of growth suggesting that furfural conversion to its alcohol is the main factor for improving the tolerance. In agreement with this, the evolution of the industrial yeast strain TMB3400 in synthetic mixtures of sugars supplemented with furfural, HMF, and acetic acid showed faster consumption of these inhibitors (Keating et al., 2006). Furthermore, the conversion of furfural to furfuryl alcohol at significantly higher rates was the solution of evolved *S. cerevisiae* and *Pichia pastoris* strains to tolerate these chemicals (Liu et al., 2005). These results suggest that detoxification of furfural and HMF can be carried out in place with yeast strains having higher ability to convert such toxic molecules.

Evolution of the industrial strain ethanol red of *S. cerevisiae* in non-detoxified spruce hydrolyzate in combination to high temperature (39°C) resulted in the selection of strains capable to convert spruce hydrolyzates into ethanol with high efficiency (Wallace-Salinas and Gorwa-Grauslund, 2013). Contrary to the resistance in evolved strains selected with furfural and HMF alone, the superior phenotype of the evolved ethanol red strains did not rely on higher reductase activities for furfural conversion, but rather on a higher thermotolerance. Different results were also observed in tolerant yeast strains obtained from evolutionary engineering using genome-shuffling technology based on large-scale population with cross-mating to generate tolerance to spent sulfite liquor (SSL) (Pinel et al., 2011). These strains were also more tolerant to higher osmolarities, elevated ethanol concentrations, and higher amounts of acetic acid than the parental strain.

Studies based on the change in gene expression using microarrays have led to the identification of redox balance and energy state of the cells as the major drivers to generate tolerance to furfural and HMF (Petersson et al., 2006; Ask et al., 2013a). From the 15 reductases which overexpression were found to improve tolerance, the overexpression of three candidate genes raised with the recognition of *ADH6* as one of the major contributors for tolerance to HMF in aerobic and anaerobic conditions (Petersson et al., 2006). It has been also demonstrated that tolerance to furfural can be increased by the overexpression of *ADH7*, the ORF *YKL071W*, and *ARI1* genes, which encode are reductases involved in furfural reduction (Heer et al., 2009; Sehnem et al., 2013). Combining the overexpression of the ADH *ADH1* with the transaldolase *TAL1* in recombinant xylose-fermenting *S. cerevisiae* improves ethanol production from lignocellulosic hydrolyzates. Most of the tolerant strains generated by these means increase furfural and HMF conversion to their less toxic alcohols. This strategy has been also effective in *E. coli*, in which the overexpression of reductases *YGHD* and *DKGA*, having NADPH-dependent furfural reductase activity, increases furfural tolerance (Miller et al., 2009).

Besides the overexpression of *TAL1*, the overexpression of some genes of the pentose phosphate pathway also increases yeast tolerance to furfural. Among them, the overexpression of *ZWF1*, *GND1*, or *RPE1* induced tolerance to furfural at concentrations that are normally toxic in the wild-type strain (Gorsich et al., 2006). These results were similar to those observed when the xylose reductase and xylitol dehydrogenase from *P. pastoris* were overexpressed in combination with overexpression of the endogenous xylulose kinase of *S. cerevisiae* (Almeida et al., 2008). On the other hand, the overexpression of *YAP1* activated the transcription of catalases genes *CTA1* and *CTT1*, enhancing the tolerance to furfural but not to HMF (Kim and Hahn, 2013), suggesting that rapid furfural consumption is associated to accumulation to reactive oxygen species.

The evolutionary engineering through genome-shuffling technology was used to increase yeast tolerance to hardwood SSL (Pinel et al., 2011). Using RNA-seq gene expression analysis, these authors found that the products of the genes *UBP7* and *ART5* (both related to ubiquitin-mediated proteolysis), *NRG1* (a stress-response transcriptional repressor), and *GDH1* (a NADPH-dependent glutamate dehydrogenase), play an important role in the tolerance to these hydrolyzates (Pinel et al., 2015). The genome-shuffling technology method was also used to increase tolerance to a combination of heat, acetic acid, and furfural stresses (Lu et al., 2012). The resulted strains showed tolerance to 0.55% (v/v) acetic acid and 0.3% (v/v) furfural at 40°C.

Tolerance to phenolics can be tackled by the expression of extracellular heterologous laccases (Lee et al., 2012). This trait can be also enhanced by heterologous expression of the gene encoding the phenyl acrylic-acid decarboxylase (PSP1), which catalyzes the decarboxylation of aromatic carboxylic acids into the corresponding vinyl derivatives (Richard et al., 2015). Overexpression of multidrug efflux pump genes *ATR1* and *FLR1*, and the TF *YAP1* also resulted in yeast resistance to coniferyl aldehyde and HMF (Alriksson et al., 2010). Tolerance to vanillin and 39°C were induced after several rounds of mutagenesis in hydrolyzates containing vanillin (Kumari and Pramanik, 2012). Chemical mutagenesis coupled with ALE using continuous cultivation in 60% (v/v) non-detoxified hydrolyzate liquor from steam-pretreated lignocellulose was successfully used to select yeast strains with improved capacity to ferment xylose from lignocellulose hydrolyzates (Smith et al., 2014). Since many of the toxic compounds affect the membrane potential, the addition of spermidine, which synchronize Ca^2+^, Na^+^, K^+^, and ATPase has also proven to induce tolerance, after disruption of the spermidine metabolism genes *OAZ1* coding for an ornithine decarboxylase (ODC) enzyme and *TPO1* coding for the polyamine transport protein (Kim et al., 2015). Changes in ergosterol composition have shown to improve tolerance to vanillin in strains overexpressing the ergosterol synthesis genes *ERG28*, *HMG1*, *MCR1*, *ERG5*, and *ERG7* (Endo et al., 2009).

### Increasing Tolerance to High Temperature and Elevated Osmolality

Adaptive laboratory evolution has been successful for selecting thermotolerant *S. cerevisiae* strains (Yona et al., 2012; Caspeta et al., 2014b). After an evolution period of 450 generations, thermotolerant yeast populations were isolated from experiments performed at 39°C (Yona et al., 2012). These populations showed a duplication in chromosome number III (ChIII) and overexpression of related genes. However, only the overexpression of some genes found in this chromosome including the TF *HCM1* and the protease *RRT12* reproduced a significant fraction of the thermotolerant phenotype in the parental strain. A similar result was found in thermotolerant yeast strains isolated from ALE experiments to 39.5°C (Caspeta et al., 2014b). In this work, a partial duplication of ChIII containing the *HCM1* gene was found. Since duplication of ChIII was lost in evolved strains, this suggests that chromosomal duplications are a temporal solution to stress (Yona et al., 2012).

Adaptive laboratory evolution experiments have also used to generate tolerance to high pH, which induced the duplication of chromosome number five (Yona et al., 2012). Remarkably, chromosomal duplications only appear in diploid cells since haploid *S. cerevisiae* populations showed segmental duplications only. In these strains, a nonsense mutation of *ERG3* proportionated 80% of the thermotolerant phenotype (Caspeta et al., 2014b). This result and the fact that ethanol tolerance can be achieved by just one overexpression suggest that complex tolerant phenotypes can be achievable by just one mutation. Remarkably, this mutation changed cellular membrane properties.

Genome-shuffling technology was used to improve yeast performance in high gravity fermentations (Liu et al., 2011). The resulted strains derived from a diploid STE2/STE2 (receptor for alpha-factor pheromone) strain increased tolerance to high osmolarity and elevated ethanol concentrations. This method was also used to generate sexual and asexual populations of *S. cerevisiae* resistant to very high gravity fermentations, elevated temperature, and high glucose concentrations (Hou, 2010). In mutants of the gene *GPD2* encoding glycerol 3-phosphate dehydrogenase subjected to three rounds of genome shuffling, a population of strains producing lower amounts of glycerol and improved tolerance to ethanol and high osmolality were able to be selected (Tao et al., 2012). These strains showed changes in FAs composition and higher accumulation of trehalose. A remarkable application of the genome-shuffling technology was the generation of both thermotolerance and ethanol tolerance in the industrial yeast strain SM-3, which were used to ferment syrups with 20% (w/v) glucose at 45°C and resists 9.5% (w/v) ethanol (Shi et al., 2009).

## Concluding Remarks

One of the major challenges for economic conversion of lignocellulose to fuel ethanol is to generate robust *S. cerevisiae* strains able to cope with inhibitory conditions while keeping proper catalytic functions for raw material conversion to ethanol. Major inhibitory conditions found in the unit operations required for the conversion processes include the accumulation of toxic chemicals generated during lignocellulose pretreatment and sugar fermentation, the high temperature that accompanied simultaneous saccharification and fermentation, and the very high osmolality and elevated solids loadings at the beginning of the fermentation. Since unification of these unit operations is desirable to reduce production costs and energy utilization, it can be expected that yeast cells will be simultaneously exposed to most of these inhibitory conditions.

Since cellular macromolecules and metabolism have evolved to sustain optimal growth rates at the prevailed natural conditions, mainly preserving genetic information and proteins/membrane functional structures, the generation of complex tolerant phenotypes for the ethanol industry will be further generated on the bases of the functions targeted by the stressors. The summarized results altogether show that major targets include cellular membrane, redox and ionic potentials, and energy metabolism, as well as protein structure – the latter of apparently minor relevance.

To establish metabolic engineering strategies for increasing yeast tolerance, it is suggested to consider the route and regulation of molecular responses following sensing, signal transduction, signal integration, and execution of cellular functions in response to environmental stresses. Results from systems biology and -omics analyses, as well as from traditional data mining, point out the relevance of the cross-regulation between the routes of yeast responses according to the different types of stress. This is part of the elasticity of cellular stress-signaling network, which is advantageous during evolutionary adaptation and in the generation of resistance to the multiple stresses found in ethanol production process.

In summary, the multiple technologies for the generation of numerous mutations, high-throughput screening, acceleration of cells adaptation and selection, laboratory evolution and engineering of TFs, and the new tools for controlling gene expression are accelerating the accumulation of basic information of CSRs, and the generation of yeast cells with desirable processing characteristics including better performance in the inhibitory conditions found in lignocellulosic ethanol production processes.

## Conflict of Interest Statement

The authors declare that the research was conducted in the absence of any commercial or financial relationships that could be construed as a potential conflict of interest.
